# Enzymology and significance of protein histidine methylation

**DOI:** 10.1016/j.jbc.2021.101130

**Published:** 2021-08-27

**Authors:** Magnus E. Jakobsson

**Affiliations:** Department of Immunotechnology, Lund University, Lund, Sweden

**Keywords:** post-translational modification (PTM), enzyme, histidine, protein methylation, proteomics, systems biology, epigenetics, 1MeH, N1-methylhistidine, 3MeH, N3-methylhistidine, 7BS, seven beta strand, Hme, histidine methylation, Hpm1, histidine protein methyltransferase 1, MT, methyltransferase, PHMT, protein histidine methyltransferase, PTM, post-translational modification, SET, (Su(var)3-9, Enhancer-of-zeste, Trithorax), SILAC, stable isotope labeling by amino acids in cell culture

## Abstract

Cells synthesize proteins using 20 standard amino acids and expand their biochemical repertoire through intricate enzyme-mediated post-translational modifications (PTMs). PTMs can either be static and represent protein editing events or be dynamically regulated as a part of a cellular response to specific stimuli. Protein histidine methylation (Hme) was an elusive PTM for over 5 decades and has only recently attracted considerable attention through discoveries concerning its enzymology, extent, and function. Here, we review the status of the Hme field and discuss the implications of Hme in physiological and cellular processes. We also review the experimental toolbox for analysis of Hme and discuss the strengths and weaknesses of different experimental approaches. The findings discussed in this review demonstrate that Hme is widespread across cells and tissues and functionally regulates key cellular processes such as cytoskeletal dynamics and protein translation. Collectively, the findings discussed here showcase Hme as a regulator of key cellular functions and highlight the regulation of this modification as an emerging field of biological research.

Post-translational modification (PTM) involves the covalent attachment of chemical groups, or peptides, to specific positions in a protein and often exerts regulatory functions (1). One prominent example is the attachment of methyl groups (-CH_3_) to the amino acid side chains of lysine, arginine, and histidine (His) as well as glutamate, glutamine, asparagine, and cysteine in addition to protein N and C termini (2, 3).

Cellular methylation is catalyzed by methyltransferase (MT) enzymes that transfer a methyl group from AdoMet to substrates (4). The human genome encodes over 200 enzymes with predicted MT activity, and these are often grouped based on predicted structural features (5). The biggest class comprises the so-called seven beta strand (7BS) MTs and the second largest group constitutes the SET (Su(var)3-9, Enhancer-of-zeste, Trithorax) domain–containing enzymes (5).

Protein histidine methylation (Hme) was first reported in 1967 (6, 7) and has attracted far less attention than methylation events on arginine and lysine, which represent key epigenetic regulators and constituents of the histone code (8). In 2018, and over 5 decades after the discovery of Hme, SETD3 was uncovered as the first human protein histidine methyltransferase (PHMT) (9, 10).

Recent exhaustive MS-based proteomics studies enable analysis of PTMs without prior affinity enrichment (11), and querying such comprehensive proteome datasets has revealed hundreds of cellular Hme events (10, 12, 13). Moreover, single modification-centric studies have linked distinct Hme events to important cellular functions such as regulation of the cytoskeleton (9, 10) and translation dynamics (14).

Here, recent findings regarding the enzymology, extent, function, and system level analyses of Hme are reflected upon and discussed. We also review the available methodology to provide technical perspectives and suggest preferred approaches for Hme analysis. We end with a prospective outlook on opportunities and development of the emerging Hme research field.

## Biochemistry of Hme

**Figure 1:**
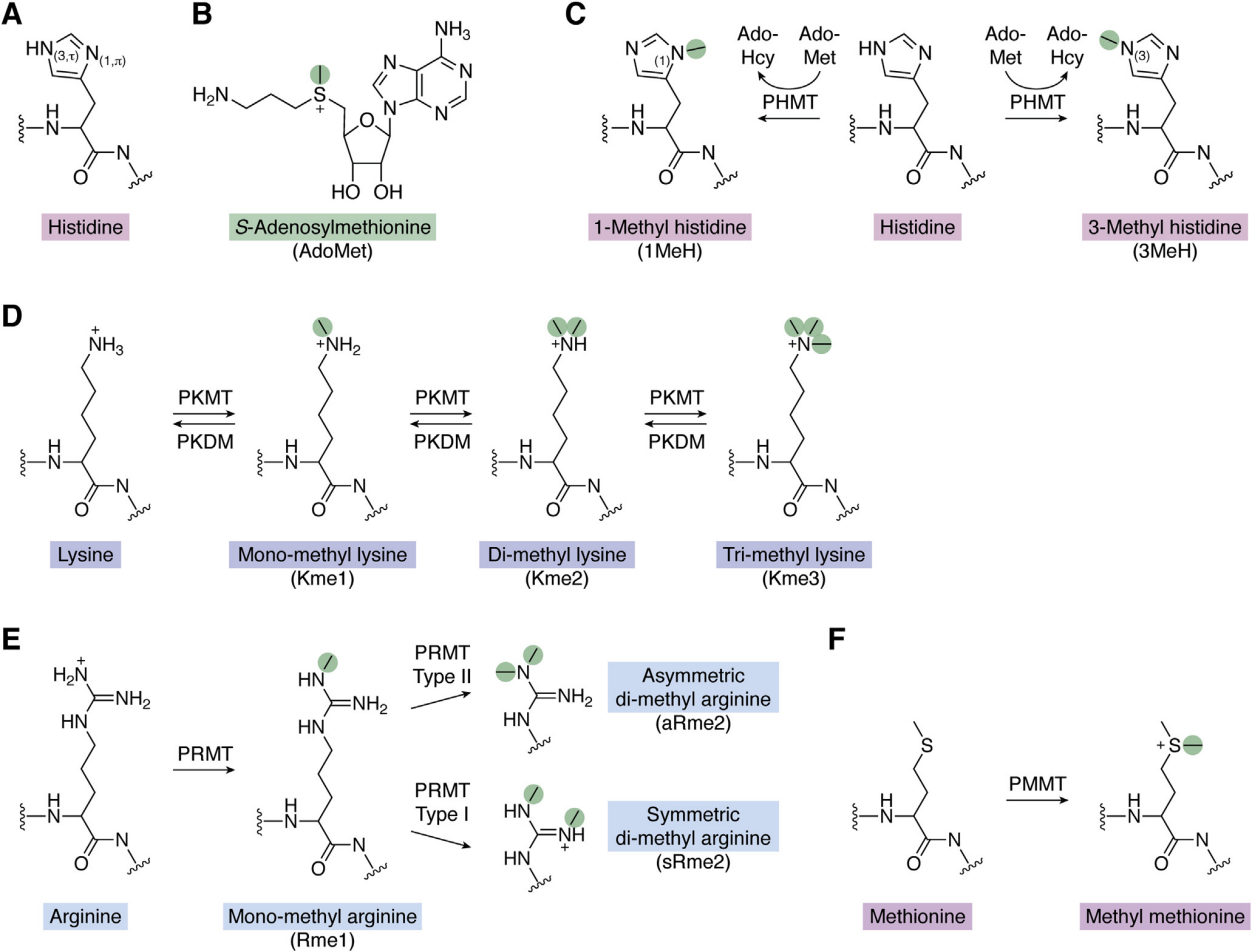
**Biochemistry of protein histidine methylation and selected key cellular protein methylation events.***A*, histidine structure and nomenclature. The imidazole ring in the histidine side chain contains two nitrogen atoms denoted as N1 (or τ) and N3 (or π). *B*, chemical structure of AdoMet. The polarized methyl group of AdoMet is highlighted (*green circle*), and the positive charge of the associated sulfonium ion is indicated. *C*, biochemistry of histidine methylation. The N1 or N3 atom of the histidine side chain can accept methyl groups through AdoMet-dependent protein histidine methyltransferase (PHMT)-mediated methylation to generate with 1-methylhistidine (1MeH) or 3-methylhistidine (3MeH) as well as the reaction byproduct AdoHcy. *D*, biochemistry of lysine methylation. Protein lysine methyltransferases (PKMT) can introduce up to three methyl groups to the ε-amino group in the side chain of lysine yielding monomethyl, dimethyl, or trimethyl lysine. In turn, the methyl groups can be removed by protein lysine demethylase (PKDM) enzymes. *E*, biochemistry of arginine methylation. Protein arginine methyltransferases (PRMT) catalyze the formation of monomethyl arginine. Subsequently, type I and type II PRMTs can catalyze symmetric dimethyl arginine and asymmetric dimethyl arginine, respectively. *F*, biochemistry of methionine methylation. Protein methionine methyltransferase (PMMT) enzymes catalyze the formation of methyl-methionine from methionine.

The amino acid His is readily recognized through its characteristic structure containing an imidazole ring. The imidazole ring contains two nitrogen atoms that are denoted as N1 (or π) and N3 (or τ) and constitutes a conjugated aromatic electron system with distinct properties (15) (Fig. 1*A*). For example, the imidazole ring can partake in interactions through so-called π-stacking with the hydrophobic residues phenylalanine, tryptophan, and tyrosine (16). Moreover, the imidazole ring has intrinsic affinity for metal cations, a feature which is frequently utilized in immobilized metal-affinity chromatography (17).

The N1 and N3 atoms of the imidazole ring can be protonated and thus exist in both a neutral and a positively charged state. The differentially protonated forms of His are in equilibrium, and their interconversion is referred to as tautomerization. Biochemically, unprotonated N1 and N3 atoms have a free electron pair with nucleophilic properties. This free electron pair can perform nucleophilic attacks on electrophiles such as the polarized methyl group of AdoMet (Fig. 1*B*), resulting in methyl transfer from AdoMet to His (18). In cells, AdoMet-dependent PHMT enzymes catalyze the generation of N1-methylhistidine (1MeH) and N3-methylhistidine (3MeH) in specific protein substrates (Fig. 1*C*). Notably, Hme bears little resemblance to other key cellular methylations such as those occurring on the guanidino group of arginine, the ε-amino group of lysine, or the sulfur in the thioether of methionine (Fig. 1, *D*–*F*).

Biochemically, Hme has several consequences. First, methylation increases the pKa of His and, consequently, the fraction protonated at the physiological pH (19). Second, 1MeH and 3MeH abrogate the ability of His to tautomerize while also increasing the void occupancy and hydrophobicity. Several enzymes make use of the inherent versatile nature of His and its chemical activity at physiological pH in active site chemistry, and one prominent example is the catalytic triad in serine proteases (20).

## Hme—early reports

During the last 2 decades, MS has emerged as the method of choice for large-scale analysis of PTMs (21). However, many PTMs were initially described and characterized in the second half of the 20th century using a range of less-sophisticated experimental approaches (22, 23, 24). Between the late 1960s and the early 1970s, independent research teams described Hme in several mammalian proteins (Table 1). In 1967, Johnson *et al.* (6) and Asatoor and Armstrong (7) described 3MeH in actin and myosin isolated from muscle tissue from a panel of animals including humans, rodents, birds, and fish. Subsequently, Johnson *et al.* (25) extended this panel of organisms, to corroborate that 3MeH in actin and myosin is widespread across the animal kingdom. Follow-up studies by Huszar and Elzinga pinpointed the site of methylation in rabbit myosin to what is now annotated as Myosin4-His756 (26) and also showed that the site was not methylated in cardiac myosin (27), suggesting that the modification is tissue specific.

**Table 1:** Early reports of mammalian protein histidine methylation

Protein	Site	Isomer (1MeH, 3MeH)	Source	Reference
Actin	ND	3MeH	Crude muscle from rabbit, cow, chicken, rat, and human	(7)
Actin	ND	3MeH	Skeletal muscle from rabbit, human, chicken, and trout	(6)
Actin and myosin	ND/ND	3MeH	Skeletal muscle from rabbit and mouse, pigeon breast, rabbit heart, cow uterus, lobster tail, and crab claw	(25)
Myosin	ND	3MeH	Adult rabbit muscle	(6)
Myosin	His756^a^	3MeH	Rabbit skeletal muscle	(26)
Histone	ND	3MeH	Duck erythrocytes	(28)
Histone	ND	ND	Human HeLa cells	(29)
MYLK2	His157	1MeH	Rabbit skeletal muscle	(30)
S100A9	His106	1MeH	Murine spleen cells	(31)

Abbreviation: ND, not determined.

a Position in myosin-4 (UniProt ID: Q28641).

Concomitant with these reports, Hme was also suggested to occur in histone proteins based on findings in avian erythrocytes (28) and human HeLa cells (29). Notably, Hme in histone proteins has not been firmly established regardless of a wealth of studies on histone PTMs using sensitive MS. These early reports of 3MeH in histone proteins may thus represent a contamination from actin, or other cellular Hme proteins.

More recent reports have described 1MeH in two additional mammalian proteins. First, Meyer *et al.* (30) reported a 1MeH-modified residue at myosin light chain kinase 2–His157 in rabbit skeletal muscle. Thereafter, Raftery *et al.* (31) found the same PTM at S100 calcium-binding protein A9 (S100A9)–His106.

Taken together, Hme has been known since the late 1960s and early reports place the PTM in a handful of mammalian proteins.

## Human PHMTs

**Figure 2:**
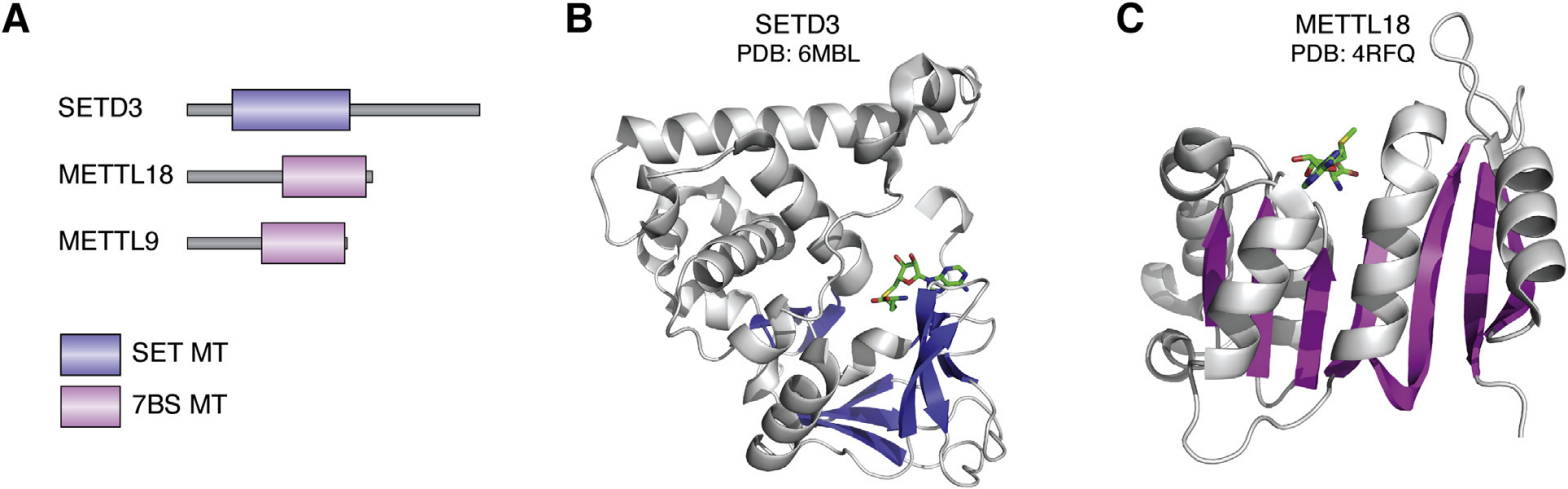
**Structural diversity of human PHMT enzymes.***A*, domain architecture of human PHMT enzymes. *B* and *C*, structures of SETD3 and METTL18. *Cartoon* representations of the MT domains and *stick* representations of the methyl donor AdoMet, or its demethylated counterpart AdoHcy, for (*B*) SETD3 (PDB ID: 6MBL, amino acids 76–320, beta strands; *light blue*) and (*C*) METTL18 (PDB ID: 4RFQ, amino acids 190–368, beta strands; *magenta*) are shown. MT, methyltransferase; PHMT, human protein histidine methyltransferase.

MT activity has been linked to five distinct human protein folds, suggesting that the enzymatic activity has arisen on several independent occasions in evolution (32). The human genome encodes over 200 predicted MT enzymes and three of these, namely SETD3, METTL18, and METTL9, have now been assigned PHMT activity (Table 2). Unlike the protein arginine MTs, the human PHMTs do not comprise a group of closely related paralogs (5, 33) (Fig. 2*A*). The SETD3 enzyme belongs to the SET domain MT family (Fig. 2*B*), whereas METTL18 and METTL9 both belong to the 7BS MT superfamily (Fig. 2*C*). Although METTL18 and METTL9 share the same 7BS fold, they are clearly unrelated enzymes. METTL18 belongs to a defined subgroup with the 7BS superfamily that has been referred to as “group J” (5) or methyltransferase family 16 (34) in the literature. In contrast to METTL18, METTL9 lacks close paralogs and can be considered an orphan enzyme sharing little sequence homology to other human 7BS MTs (5).

**Table 2:** PHMT enzymes in humans and yeast

Protein	Alt name	Organism	Specificity	Isomer	Function	Reference
SETD3	C14orf154	Hs	Actin–His73	3MeH	Actin polymerization, smooth muscle contractility	(9, 10)
METTL18	ASTP2, C1orf156	Hs	RPL3–His245	3MeH	Translation dynamics, pre-rRNA processing, polysome formation	(14)
METTL9	DREV, CGI-81	Hs	HxH, x = A, N, G, S, or T	1MeH	Zn binding, respiration	(59, 60)
YIL110W	Hpm1	Sc	Rpl3–His243	3MeH	rRNA processing, ribosome assembly	(52, 55)

Abbreviations: Hs, *Homo sapiens*; Sc, *Saccharomyces cerevisiae*.

Taken together, PHMT activity has arisen on at least three independent occasions in evolution and can develop on different proteins folds, that is, on both SET and 7BS MT domains.

## SETD3-mediated actin methylation

Actin is a dynamic constituent of the cytoskeleton and can exist in both a monomeric and a polymeric filamentous form (35). Polymerization dynamics are driven by ATP and enable cells to apply force, driving, for example, cell migration (35).

After the initial reports revealing that actin contains a 3MeH residue, several studies pointed to His73 as the site of methylation (36, 37, 38), but the responsible MT was elusive for a long period. In 1987, Vijayasarathy and Narasinga Rao described a protocol for the enrichment of an actin-specific PHMT activity from rabbit skeletal muscle (39) and later also detailed protocols for assaying actin MT activity (40, 41). Lacking access to today’s sensitive MS-based discovery proteomics workflows, they never identified the enzyme. Almost 3 decades later, in 2018, Kwiatkowski *et al.* (9) used a similar multistep chromatography approach and MS-based identification of enriched proteins to uncover SETD3 as the actin–His73 PHMT. Concomitantly, Wilkinson *et al.* (10) independently revealed the actin-specific PHMT activity of SETD3 and extensively characterized the biological and physiological consequences of Hme in actin. They elegantly demonstrated that ACTB–His73 likely represents the only physiological substrate for SETD3 through a comprehensive methylproteomic analysis of gene-targeted HeLa cells. A quantitative comparison of methylation in SETD3 KO cells revealed loss of only actin–His73 methylation among over 900 identified protein methylation sites. Moreover, from studies using a KO mouse model, they conclude that ACTB–His73 methylation occurs in a wide range of cells and tissues and is exclusively dependent on SETD3 *in vivo*. Functionally, the modification was shown to reduce the rate of nucleotide-exchange for actin monomers, which promotes the assembly of actin filaments. In line with these findings, Nyman *et al.* (42) reported that actin-His73Ala mutants isolated from budding yeast display increased ATP exchange rate as well as inhibited polymer formation. Importantly, budding yeast lacks SETD3 and the WT actin control in the study was unmethylated at His73. Thus, the study implies a general, and methylation-independent, role of His73 in actin polymerization. Notably, the physiological importance of these *in vitro* findings is difficult to foresee because replacing endogenous actin with a range of actin-His73 mutants does not result in a clear phenotype in budding yeast, but the corresponding mutations clearly alter *in vitro* properties of actin (43).

The physiological consequences of actin-His73 methylation have been explored on both a cellular and organismal level. Kwiatkowski *et al.* (9) reported reduced levels of F-Actin in SETD3-deficient HAP-1 cells and that actin from KO cells displays reduced stability at physiologically relevant temperatures. They also found that SETD3 KO cells consume more glucose and produce more lactate than the WT, suggesting that metabolism may be rewired. Wilkinson *et al.* (10) explored the role of SETD3 in a physiological context using a KO mouse model. Female mice lacking SETD3 suffered from primary dystocia and reduced litter size. Analogously, human uterine smooth muscle SETD3 KO cells were shown to display impaired signal-induced contraction.

SETD3 is conserved in the animal kingdom and present in most multicellular organisms including mammals, insects, and plants, but absent in budding yeast (Table 3). This distribution matches well with organisms with reported actin-His73 methylation (Table 1), which is also absent in budding yeast (44). Taken together, the above reports highlight Hme as a regulator of actin dynamics and with physiological consequences related to muscle function.

**Table 3:** Human PHMT enzymes and their orthologs in common model organisms

Protein query	UniProt identifier	Refseq accession #	Putative orthologs (expect value^a^)			
			*Drosophila melanogaster*	*Caenorhabditis elegans*	*Arabidopsis thaliana*	*Saccharomyces cerevisiae*
SETD3	Q86TU7	NP_115609.2	NP_727144.1 (4e-72)	NP_497604.2 (2e-37)	NP_172856.1 (8e-27)	-
METTL9	Q9H1A3	NP_057109.3	NP_611846.1 (9e-39)	NP_508880.2 (6e-55)	-	-
METTL18	O95568	NP_219486	NP_608740.2 (1e-45)	NP_497707.1 (3e-30)	NP_567417.1 (1e-29)	NP_012156.1 (3e-12)

a Using NCBI's protein BLAST function to query nonredundant sequences from all organisms.

Before the discovery of SETD3 as a PHMT, SET domain MTs had exclusively been shown to target lysine residues in substrate proteins (45). SETD3 was initially also claimed to be a histone H3–Lys4–specific MT (46), but recent studies have reported and corroborated (9, 10) actin–His73 as the physiological substrate. The somewhat unanticipated PHMT activity of SETD3 instigated several studies to comprehend its structure–function relationship. Cocrystallization studies of actin–SETD3 revealed that the proteins undergo striking conformational changes upon interaction and particularly a rotation of the actin–His73 imidazole ring (47). Interestingly, these structural studies enabled the rational design of an SETD3 double-point mutant with a preference for lysine over His methylation (48). More recently, it was unexpectedly demonstrated that SETD3 can methylate methionine residues artificially introduced at the position corresponding to actin–His73 (49). This supports a model where SETD3 recognizes a folded actin substrate and mediates methyl transfer to His73, or other nucleophilic amino acid artificially introduced in this position.

In summary, studies on SETD3 biochemistry have revealed several surprises, and conflicting reports, regarding its enzymatic activity. Its activity toward actin–His73 has been independently validated (9, 10) using a range of complementary experimental techniques, whereas its role as a histone H3-Lys4 MT has not been confirmed in recent studies (10).

## METTL18-mediated RPL3 methylation

RPL3 constitutes a part of the large ribosomal subunit and is conserved in the eukaryotic kingdom (50, 51). In 2010, Webb *et al.* (52) in the Clarke lab first described Hme in budding yeast and showed that RPL3 contains a 3MeH modification at His243. They further demonstrated that it is exclusively dependent on an MT encoded by the YIL110W gene, which was given the descriptive name histidine protein methyltransferase 1 (Hpm1). Hpm1 belongs to the 7BS MT superfamily and human METTL18 represents a clear sequence ortholog (53) (Table 2). Malecki *et al.* (14) recently reported that METTL18 also represents a functional homolog of Hpm1 and catalyzes methylation of the corresponding site in human RPL3, that is, RPL3–His245.

METTL18 belongs to a defined subgroup of enzymes within the 7BS superfamily (5, 54) that was initially proposed to exclusively comprise enzymes with protein lysine MT activity (53). Therefore, the identification of METTL18 as a PHMT was somewhat surprising. Collectively, the studies on METTL18 and SETD3 highlight that PHMT activity can arise on different structural MT folds (*i.e.*, SET and 7BS) and that minor changes in their activity sites can sway the amino acid substrate specificity from lysine to His. In turn, this highlights the difficulty in predicting MT specificity based on sequence information.

The functional consequences of RPL3 Hme are related to ribosome assembly and function. In budding yeast, loss of methylation causes defects in rRNA processing (55) and an increased sensitivity to translation stress (52). Human HAP-1 METTL18 KO cells display a similar phenotype with altered pre-rRNA processing and decreased polysome formation (14). Malecki *et al.* also explored the role of METTL18-mediated methylation in translation dynamics. They devised a sophisticated ribosome foot printing approach to globally explore codon-specific translation kinetics. The approach relies on assessing the occupancy of codons in the ribosome acceptor (A)-site, that is, the site where the ribosome samples cellular aminoacyl-tRNA complexes during translation, and on the assumption that the frequency of a codon in the A-site reflects it rate of translation (56). Intriguingly, METTL18-deficient cells displayed altered codon-specific changes in mRNA translation and, most notably, a striking decrease in the translation rate of GAA (glutamate) (14). This supports a model where methylation of RPL3-His245 may regulate protein synthesis through mRNA translation to shape the proteome.

Few studies have yet focused on the physiological roles of METTL18 and its link to diseases. However, METTL18 gene amplification has been linked to a range of cancers including bladder urothelial carcinoma, liver hepatocellular carcinoma, breast invasive carcinoma, as well as lung adenocarcinoma in large-scale sequencing studies (57). More recently, the protein was reported as elevated in plasma from patients with colorectal cancer (58). Collectively, these reports suggest the protein may have clinical utility as a cancer biomarker.

## Pervasive 1MeH by METTL9

In contrast to SETD3 and METTL18, METTL9 is an orphan MT without clear paralogs (5). Of the human PHMTs, METTL9 is also the least conserved across species. It is present in nematodes and mammals but absent in plants and yeast (Table 2), suggesting that the enzyme catalyzes a function specific to organisms in the animal kingdom.

Davydova *et al.* (59) recently uncovered METTL9 as the first 1MeH-specific enzyme, and third human PHMT, using a wide range of biochemical and cellular assays, and this finding has been independently validated (60). The enzyme was shown to catalyze the generation of 1MeH in the C-terminal His residue of a linear sequence motifs corresponding to HxH, where “x” represents a small amino acid such as alanine, asparagine, glycine, serine, or threonine. This degenerate motif is present close to 3000 occasions across the human proteome. Notably, the long-known S100A9–His106 site is present in such a HxH motif, and METTL9-deficient cells were indeed shown to lack 1MeH at the site. The authors also performed a global analysis of the Hme-to-His ratio and estimated that the human proteome contains Hme1 corresponding to 125 fully modified sites, and the bulk of these are dependent on the METTL9 enzyme (59).

Few studies have yet focused on the physiological implications of METTL9 and its links to disease. However, independent genetic studies have linked chromosomal deletion of a segment encompassing METTL9, as well as the IGSF6 and OTOA genes, to colonic hypoganglionosis (61) and hearing loss (62).

The molecular functions of pervasive METTL9-mediated 1MeH otherwise appear mild under nonstressed conditions. For example, METTL9 KO mice do not display a strong and apparent phenotype (59). However, deletion of the MT in human HAP-1 cells led to a complex phenotype with aberrations in nucleic acid metabolism and vesicle-mediated processes (12). In line with these results, recent spatial proteomics studies place METTL9 in a secretory neighborhood encompassing membrane-associated structures such as the endoplasmic reticulum, Golgi, and plasma membrane across a range of cell lines (63). Collectively, this may suggest a regulatory function of METTL9 in membrane-linked or vesicle-mediated processes.

The degree of methylation at METTL9 target sites raises an interesting question regarding its regulation. Clearly, HxH motifs are frequently not fully methylated in cells and the degree of methylation can be increased by METTL9 (59). This supports a model where the modification may be induced, and tuned, in response to certain stimuli through the regulation of METTL9, and potentially yet unidentified PHMTs and protein His demethylases. The dynamic nature of Hme has yet not been evaluated, but it can be experimentally explored by assessing the relative turnover of Hme-modified protein species and corresponding bulk protein through pulse-chase experiments relying on a combination of dynamic (64, 65) and heavy methyl stable isotope labeling by amino acids in cell culture (SILAC) (66). The potential regulation of pervasive 1MeH, particularly, in the context of HxH motifs, will likely be explored in future work.

## Protein His methylomics

Nowadays, large-scale analysis of PTMs is most frequently performed using MS-based workflows, where specific PTM-bearing peptides are enriched using affinity agents before analysis (21, 67). For Hme, there are no established affinity agents available to enrich modified peptides or proteins. As an alternative approach, three recent studies (10, 12, 13) have taken advantage of the throughput and sensitivity of modern MS and queried comprehensive proteomics datasets for Hme (Table 4).

**Table 4:** Hme events identified in proteomics studies

Source	Hme sites	Reference
HeLa	299	(12)
HeLa	181	(10)
HEK293	80	(12)
HEK293	103	(13)
A547	65	(12)
HCT116	89	(12)
MCF7	65	(12)
Sy5Y	98	(12)
Prostate tissue	46	(12)
Liver tissue	30	(12)
Colon tissue	47	(12)

**Figure 3:**
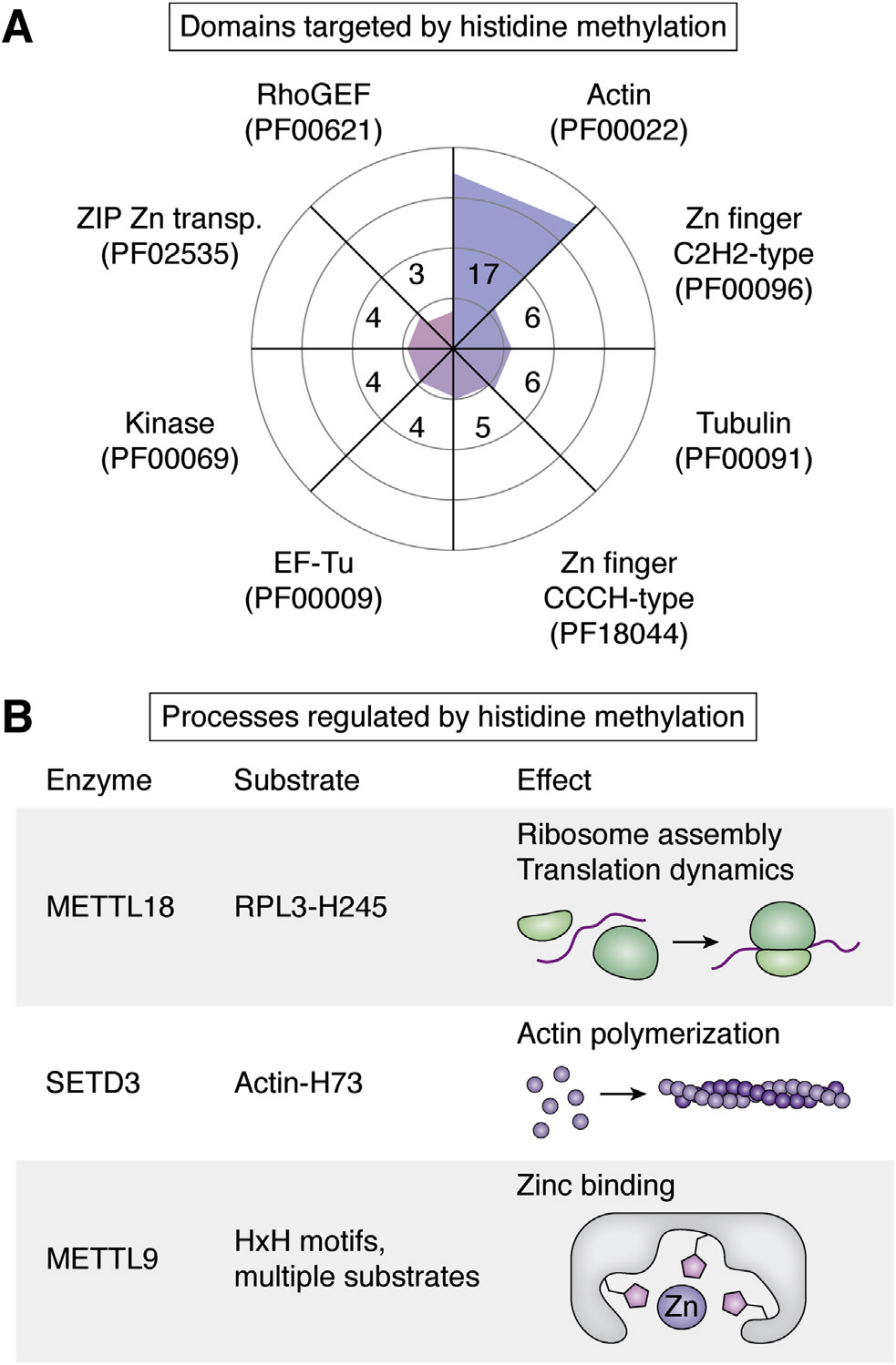
**Targets and functions of histidine methylation.***A*, domains targeted by histidine methylation. Radar plot of enriched Pfam domains for Hme in comprehensive HeLa proteomics data is shown. *B*, molecular functions regulated by histidine methylation. The molecular functions of well-characterized histidine methylation events as well as the responsible enzymes and targeted sites are indicated.

Collectively, these proteomics studies provide evidence of over 1000 cellular Hme sites and demonstrate that the modification is abundant and widespread across human cells and tissues. The yet most comprehensive Hme-ome from a single cell type stems from HeLa cells and comprises 299 unique sites (12) (Table 4). In this dataset, Hme is clearly overrepresented in actin and zinc-binding domains (Fig. 3*A*). In contrast, Rme is often reported as overrepresented in RNA-recognition domains (12, 68, 69) and Kme in histone protein domains (12, 13).

The enrichment of zinc-binding domains for Hme is particularly interesting because His can coordinate cation binding. In proteins, zinc is frequently coordinated by His-containing motifs (70, 71). Hme was also recently found overrepresented in such a primary sequence context with additional His residues in the −4, −2, +2, and +4 positions (12). This motif corresponds well to the METTL9 target motif, and indeed, methylation of HxH motifs in peptides modulate the affinity for zinc ions (59) (Fig. 3*B*).

Most reports interpreting PTM data rely on the assumption that modifications are enzyme-regulated and nonenzymatic PTM is a concept that is largely overlooked in biological research. For Hme, nonenzymatic reactions may not be negligible. It is well established that the major cellular methyl donor AdoMet can partake in nonenzymatic methylation (72) and the nucleophilic nature of the His side chain may engage in reactions with AdoMet in the absence of enzymes at the physiological pH (18). For SETD3, a recent study suggests that nonenzymatic methylation is slow compared with enzyme-catalyzed modification (18). However, the relative contribution of nonenzymatic Hme to the Hme-ome, and its potential function, remains to be investigated in detail.

## PTM crosstalk

The combined action, or interdependence, of PTMs is often referred to as cross-talk or interplay (73). Conceptually, crosstalk can occur through two distinct mechanisms. First, PTMs with a high site occupancy that occur on the same amino acid are frequently mutually exclusive and can directly compete. Second, PTMs can prime for, or inhibit, further modification at adjacent sites.

Intricate PTM crosstalk is a central concept in the field of epigenetics and represents an integral part of the “histone code” hypothesis (74). Recently, similar “PTM codes” have also have been proposed to regulate, for example, chaperone protein function through a “chaperone code” (75, 76) and mRNA translation through an “eEF1A code” (77, 78, 79).

Aside from being methylated, the N1 and N3 positions of the His imidazole ring can be phosphorylated (80). His phosphorylation and methylation are mutually exclusive and cannot occur on the same residue. Moreover, His phosphorylation is a labile PTM that is lost during standard phosphoproteomics workflows and is thus scientifically underexplored (80). New tailored workflows for His phosphorylation proteomics enable both stabilization and affinity enrichment of the PTM (81). Future studies will likely explore the overlap of His phosphorylation and methylation sites and elucidate whether the PTMs interplay through competition.

Colocalization of Hme and phosphorylation of serine, threonine, and tyrosine were recently assessed in a system level methylproteomics analysis of human cells and tissues (12). The study revealed over 300 Hme sites colocalizing with phosphorylation that were shown to be of likely high functional importance, when evaluated using a recent machine learning–based functional scoring approach (82).

PTM competition and colocalization require different experimental approaches for analysis by MS. As competition intrinsically occurs between mutually exclusive PTMs, it is best assessed through parallel affinity enrichment workflows of peptides from single samples. In contrast, colocalization is best analyzed through workflows involving sequential affinity enrichment to extract peptides bearing multiple modifications (83, 84).

Taken together, recent studies suggest that Hme may crosstalk with phosphorylation. Along with the development of robust workflows for His PTM analyses, the role of Hme in PTM interplay will likely be further explored.

## Challenges in analyzing Hme

Despite the recent tremendous advances in comprehending the enzymology, extent, and function of Hme, its analysis is still associated with several challenges. Although modern MS displays impressive sensitivity (85, 86), it cannot readily distinguish peptide isomers with identical mass, such as 1MeH and 3MeH. Moreover, all naturally occurring monomethylation events as well as the single amino acid substitutions valine-to-isoleucine and glycine-to-alanine give rise to identical mass shifts (66). Collectively, this renders MS-based methylation site identification challenging and the process is associated with a notable increased false discovery rate (87).

One approach to distinguish protein methylation events and mass shifts introduced by SNPs is so-called heavy methyl SILAC (66, 88). Heavy methyl SILAC involves growing cells in the presence of Met with an isotopically heavy methyl group. In cells, Met is converted into AdoMet and the heavy labeled methyl group is subsequently incorporated into proteins by MT enzymes. Consequently, methylated proteins get a mass shift, which is readily distinguishable from valine-to-isoleucine and glycine-to-alanine mutations.

To pinpoint which site in a peptide that bears methyl group, most MS data search algorithms, such as the widely used MaxQuant (89), allow searching for several methylation in parallel and report a localization probability score that can be used to filter high-confidence methylation events, an approach widely applied in phosphoproteomics (90, 91). It was recently reported that Hme-bearing peptides can give rise to a specific immonium ion in tandem mass spectra upon fragmentation (12). The Hme immonium ion has a mass of 124.087 atomic mass units, a region with low background signal in mass spectra (92). Inspection of mass spectra for this immonium ion can corroborate Hme events and enable Hme bearing peptides to be distinguished from those with SNPs or other monomethylation events.

The isomers 1MeH and 3MeH have identical mass and cannot be distinguished by MS alone. However, they display different chromatographic properties (93) and can thus be distinguished by chromatography using synthetic standards. Likely, peptides bearing the different isomers can be separated and distinguished using a combination of high-resolution liquid chromatography coupled to MS and synthetic heavy isotope–labeled peptide standards, but this approach has not yet been tested.

Another approach to distinguish 1MeH and 3MeH would involve enrichment using isomer-specific affinity agents before MS analysis. Such affinity agents are not yet available, but recent reports have demonstrated the generation of molecularly imprinted polymers to enrich His phosphorylated peptides for proteomics (81), an approach also applicable to Hme. The generation of polyclonal antibodies through rabbit immunization has also been proven feasible for His phosphorylation (94, 95), but also not yet applied to Hme. As an alternative, recombinant antibodies that distinguish the isomers may be selected from phage-displayed recombinant antibody libraries. This approach has been successfully applied to generate antibodies distinguishing sulfo-tyrosine and phospho-tyrosine (96), but not yet been applied to His PTMs. Taken together, a range of methods are available to generate affinity binders to enrich Hme for proteomics applications, and future research efforts will likely be made in this direction.

PTMs can also be analyzed by targeted MS, where an instrument is programmed to search for specific masses, such as those corresponding to PTM-bearing peptides (97, 98, 99). Chemically stable PTMs are well suited for such analysis and, notably, methylations are generally considered stable and can be analyzed using a range of MS acquisition and fragmentation approaches (100, 101), which is a distinct advantage compared with labile glycosylations (102). Hme may thus be analyzed using large-scale targeted MS approaches. To this end, the recent Hme proteomics studies collectively comprise a resource of over 1000 cellular Hme sites that may be used for the design of targeted MS methods, allowing quantitation of Hme events across cellular conditions.

Misidentification and erroneous reports of methylation events and the responsible enzymes can propagate and potentially set back the development of research. For lysine methylation enzymology, the Jeltsch lab has proposed an excellent set of experimental guidelines for the research community (103). These include (i) using both MT target site mutants and (ii) inactivating MT mutants as controls in *in vitro* assays, as well as (iii) quantitative assessment of enzymatic activities, including stoichiometry, using, for example, MS. These guidelines are preferably also applied in PHMT research. If applied, they would likely have circumvented the initial and plausible misidentification of SETD3 as a histone MT (10, 46).

## Conclusion and outlook

**Figure 4:**
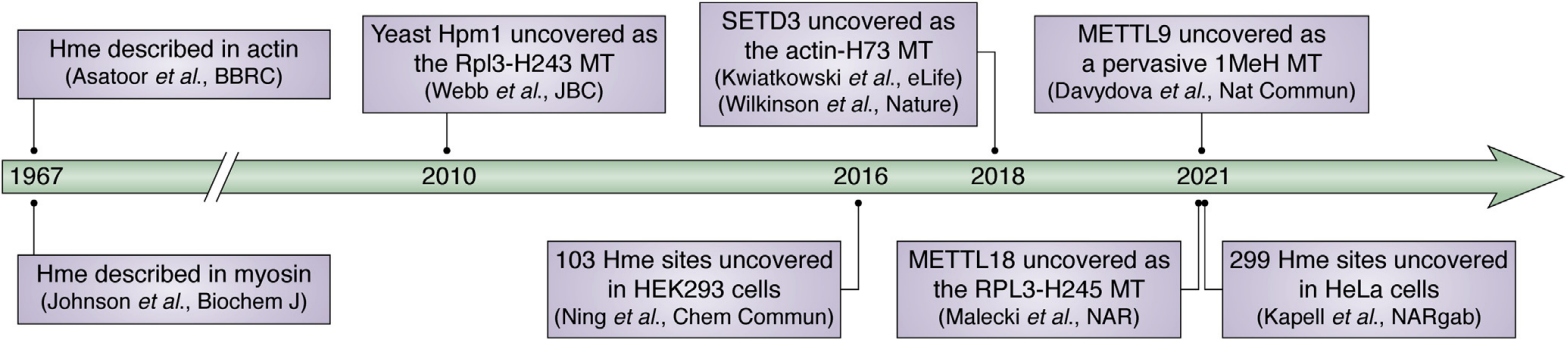
**Hallmark studies on protein histidine methylation**.

Despite being known for long, Hme has only recently gained considerable attention and it is now clear that the PTM is enzymatically regulated and widespread across tissues, cells, and subcellular structures (Fig. 4). In the near future, significant efforts to further comprehend the regulation as well as the molecular and physiological functions of Hme will likely be made. MS-based analyses are anticipated to play a key role, and to analyze Hme in scale, new affinity agents will likely be generated, potentially that discriminate 1MeH and 3MeH. Although three human PHMTs have now been identified, the enzymes that introduce 1MeH in myosin light chain kinase 2 and 3MeH in myosin, as well as the numerous sites identified in recent proteomics studies, are yet elusive. Moreover, “erasing” demethylating enzymes and “reader” protein domains, which can tune Hme levels and mediate functional outputs, remain to be discovered. Finally, PTM crosstalk is currently being explored in several processes beyond chromatin biology and epigenetics. Recent evidence suggests that Hme colocalizes with functionally important phosphorylation sites and the role of Hme in PTM interplay will likely be further explored in the coming years.

## Conflict of interest

The author declares that he has no conflicts of interest with the contents of this article.
