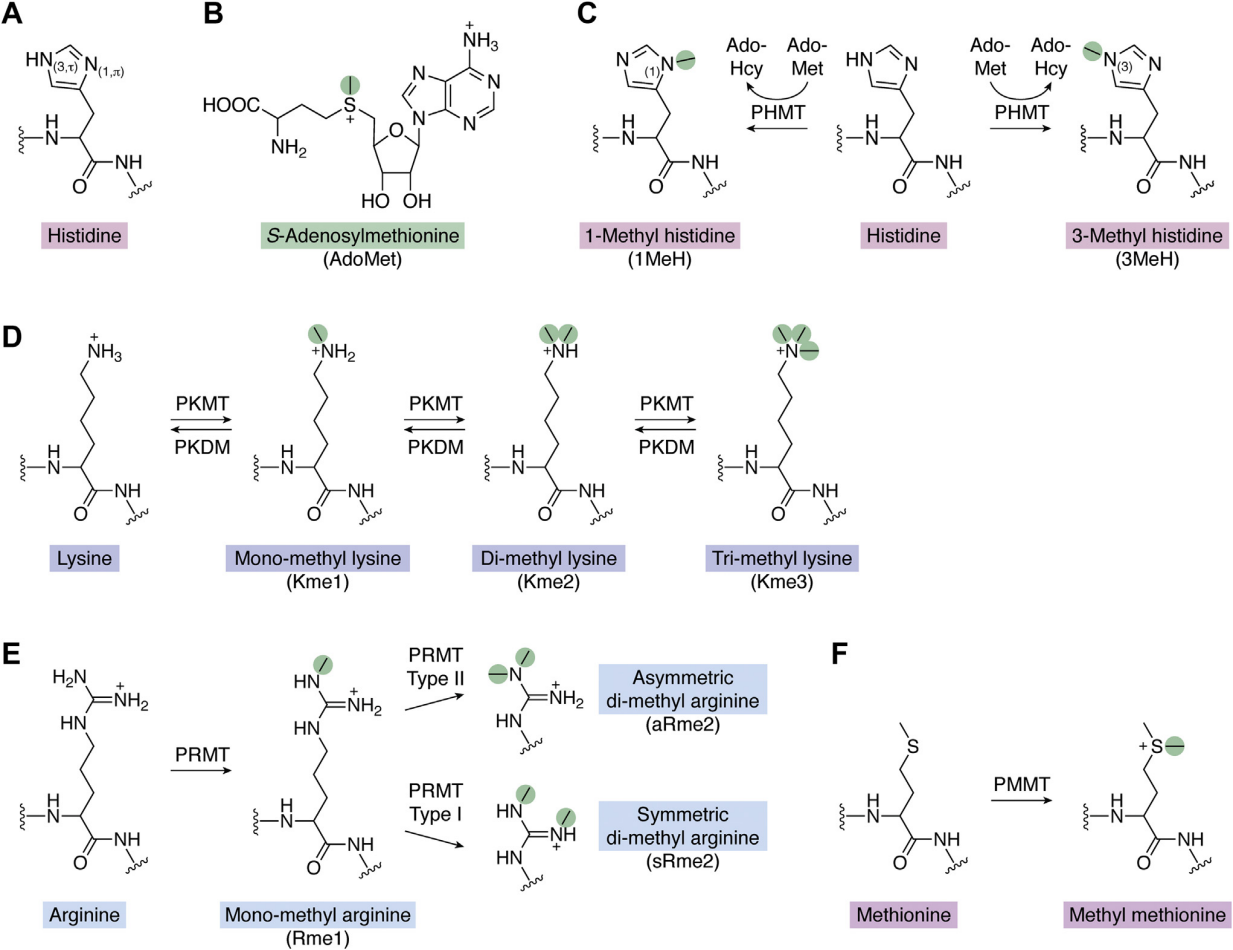# Correction: Enzymology and significance of protein histidine methylation

**DOI:** 10.1016/j.jbc.2021.101439

**Published:** 2021-11-26

**Authors:** Magnus E. Jakobsson

During Figure 1 preparation the carboxyl group in the AdoMet structure on panel B were accidentally not included. Peptide bonds were missing a hydrogen on several panels.

**Figure:**